# Risk of recurrent spontaneous preterm birth among those with persistent cervical HPV infection

**DOI:** 10.21203/rs.3.rs-9861232/v1

**Published:** 2026-06-02

**Authors:** Jessica Weng, Yan Li, Deondre Jordan, Maryam Shahi, Andrew Norgan, Elizabeth Ann L. Enninga, Margaret Long, Alyssa M. Larish

**Affiliations:** Mayo Clinic; Mayo Clinic; Mayo Clinic; Mayo Clinic; Mayo Clinic; Mayo Clinic; Mayo Clinic; Mayo Clinic

**Keywords:** Premature Birth, Preterm Premature Rupture of the Membranes, Obstetric Labor, Premature, Human Papillomavirus Viruses

## Abstract

**Background:**

Preterm premature rupture of membranes (PPROM) and preterm labor (PTL) are adverse obstetrical outcomes that are strongly associated with cervical high-risk human papilloma virus (cHPV) infection at the time of pregnancy. Persistent cHPV may lead to oncogenic changes in the cervico-vaginal tract and can cause inflammation in gestational tissues, but it is unknown how persistent cHPV infection may affect obstetrical outcomes. The aim of this study is to evaluate whether persistent cHPV infection is associated with recurrent PPROM or PTL among patients with a prior spontaneous preterm birth due to PPROM/PTL, and to examine clinical and pathologic characteristics of cHPV infection in this population.

**Methods:**

We conducted a retrospective cohort study of patients delivering at Mayo Clinic Rochester or Mayo Clinic Health System (5/1/2018–12/31/2023). Eligible patients had ≥ 1 spontaneous preterm birth due to PPROM/PTL and positive cHPV testing. Demographics and pregnancy outcomes from index and subsequent pregnancies were abstracted. Persistent cHPV was defined as > 1 pregnancy with positive cHPV testing.

**Results:**

Of 191 index pregnancies, 90 (47.1%) and 30 (33.3%) had one and two subsequent pregnancies, respectively. Patients were predominantly White (91.3%), non-Hispanic (94.2%), non-smokers (72.0%), and 34.8% were unvaccinated for HPV. Approximately one-third (31%) experienced recurrent PPROM/PTL in the second pregnancy, with recurrence in 40.7% of those with persistent cHPV and 27.4% without (p = 0.69). Excisional procedures occurred before 12.0% of index pregnancies and 20–24% of subsequent pregnancies; recurrence of PTL/PPROM remained high regardless of procedure history. HPV strain data were available for 132 index pregnancies, and types 16 and 18 were more likely to persist into a subsequent pregnancy (p = 0.0043). CIN persistence occurred at similar rates in patients with and without persistent cHPV infection. Placental inflammation rates were high in both patients with persistent and non-persistent cHPV.

**Conclusion:**

Recurrent PPROM/PTL, CIN persistence, and placental inflammation were common; a finding independent of persistent cHPV infection, excisional procedure history, or HPV vaccination status. These findings suggest that factors other than cHPV persistence may play a larger role in recurrent preterm birth. Larger studies with higher cHPV prevalence and subsequent pregnancies are needed to provide clarity.

## Background

Cervical human papilloma virus (cHPV) infections have long been known to lead to oncogenic changes in the human genito-urinary tract, but their role in recurrent adverse obstetric outcomes is just beginning to be elicited (1–3). Of particular interest are preterm labor (PTL) and preterm prelabor rupture of membranes (PPROM), which maintain a strong association with cHPV infection (3–12). A recent meta-analysis of 38 studies noted an association between active cHPV infections, PPROM and PTL, with lesser associations to other adverse pregnancy outcomes such as intrauterine growth restriction, small for gestational age birth weight, and fetal demise (3). Spontaneous preterm birth is one of the leading causes of perinatal morbidity and mortality worldwide (13). PPROM, which often leads to preterm birth (PTB), is associated with increased risk of chorioamnionitis and adverse neonatal outcomes associated with prematurity (14). Most notably, a recent meta-analysis demonstrated an association between persistent cHPV, specifically persistent type HPV-16 and HPV-18 infection, and PTL, independent of prior cervical excisional procedures (3). Finally, this association was also found on a recent population-based cohort study using a comprehensive multivariable analysis strategy, noting that cHPV infection remained associated with PPROM when adjusting for numerous factors, including a history of PPROM, cervical conization, drug use or smoking, parity, ethnicity, and insurance (2).

It has been hypothesized that ascending cervical infection from HPV can potentially lead to fetal membrane and placental inflammation, resulting in PPROM (1–3, 15–21). In fact, patients with adverse pregnancy outcomes, including those who underwent cesarean delivery with unruptured membranes, have demonstrated placental HPV positivity by multiple methods, including HPV DNA PCR, in-situ hybridization to high-risk HPV DNA, immunohistochemistry for L1 viral capsid, western blotting, and transmission electron microscopy (10, 22, 23). These discoveries generated a research interest in defining the role cHPV plays in contributing to recurrent adverse obstetric outcomes, most notably in PPROM and PTL.

While cHPV has been implicated as a contributor to the pathogenesis of adverse obstetric outcomes there is still an urgent need to determine whether persistence of cHPV infection is associated with an increased recurrence risk of PTL or PPROM. We hypothesize that persistence of cHPV virus increases the risk of recurrent PTL or PPROM. Thus, our aims are to determine the risk of recurrent adverse pregnancy outcomes among patients with cHPV infection and spontaneous PTB due to PTL or PPROM, and second, to examine the cervical histology and placental pathology of patients with cHPV infection and adverse pregnancy outcomes.

## Methods

### Study Cohort

**Figure 1:**
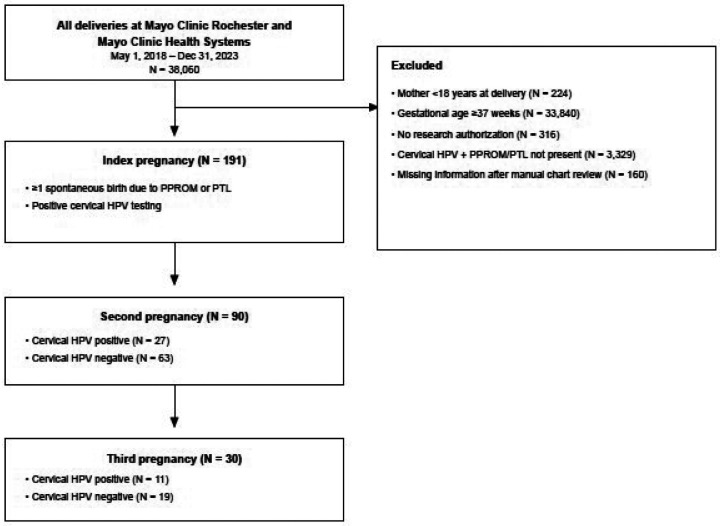
Study flow diagram. PPROM: preterm premature rupture of membranes; PTL: preterm labor; HPV: human papillomavirus.

Authorization for this study was granted by the institutional review board (IRB) of Mayo Clinic, Rochester, MN, USA, or Mayo Clinic Health System (IRB 21–005983). Patients that delivered between 5/1/2018 and 12/31/2023 were considered eligible if they had both one or more spontaneous births due to PPROM/PTL and a positive cHPV polymerase chain reaction (PCR) test any time before or during index pregnancy. Patients with any delivery < 37 weeks of gestation were included in the index pregnancy by ICD 9/10 codes for PPROM/PTL; there were no gestational age restrictions for subsequent pregnancies to be included. Patients were identified through a retrospective query of the Mayo Clinic electronic medical record database. Antenatal HPV cytology and colposcopy from this group were between 11/5/2003 and 12/7/2021. HPV status was primarily determined through internal laboratory reports. HPV laboratory tests tested for high risk strains 16, 18, and other high-risk strains. To ensure a comprehensive dataset, we performed a manual chart review of external records and scanned documents within the electronic medical records to capture results from external institutions. Patients without a definitive laboratory or pathology report were excluded from the primary analysis for the index pregnancy. Antenatal was defined as a result closest and prior to the index, second, or third pregnancy. cHPV persistence was defined as a positive PCR test following the index pregnancy and prior to the conclusion of a subsequent pregnancy. Guidelines for care and testing of cHPV at Mayo Clinic followed national guidelines per the American Society for Colposcopy and Cervical Pathology (24–26). Electronic health records were used to gather data for retrospective chart review, which was completed by a medical student and staff obstetrician with a percent median agreement of 100% and a median Cohen’s ĸ of 0.9 and 1 for continuous and binary variables, respectively. Demographics, pregnancy history and outcomes, cervical cytology or procedures, and placental pathology from the index and subsequent pregnancies were abstracted. cHPV persistence was defined as more than one pregnancy with positive cHPV testing. The study cohort selection is summarized in Fig. 1. Patients with a prior spontaneous PTB were managed per institution guidelines outlined in **Appendix 1**.

### Data Analysis

Primary outcomes included recurrent spontaneous PTB due to PPROM and PTL stratified by persistence of cHPV infection. Secondary outcomes included persistence of CIN and rates PPROM/PTL among patients with cervical excisional procedures. Information gathered from data abstraction was collected in REDCap (27), a secure database, and statistical analyses were performed using SAS software version 9.4 (SAS Institute Inc., Cary, NC, USA). Categorical variables included race, ethnicity, insurance type, HPV vaccination status, route of delivery, group type, fetal growth restriction, multiple gestation, LEEP or cone, and colposcopy (nominal), and educational level, smoking status, cytology, HPV type, and biopsy results (ordinal). Continuous variable included gestational age at delivery, gravidity, and parity. Descriptive statistics were reported with counts and frequencies for categorical variables and means and standard deviations for continuous variables. Subgroup comparisons were performed to generate hypotheses. Kruskal-Wallis tests were used for continuous variables, and Fisher’s test and chi-squared test were applied to categorical variables. A p-value of < 0.05 was considered statistically significant.

## Results

### Patient characteristics and obstetrical information

191 patients were found to be eligible based on one or more spontaneous births due to PPROM/PTL and positive cHPV testing from 5/1/2018– 12/31/2023. Out of the whole cohort, 90 and 30 patients had second and third subsequent pregnancies, respectively (Fig. 1). Most patients were White (91.3%) and non-Hispanic (94.2%), with private (55.0%) or Medicaid (41.4%) insurance. Most patients had a high school diploma and beyond (92.6%). 51.3% of patients were current (28.0%) or former (23.3%) smokers. HPV vaccination at the index pregnancy was equally distributed between started (32.6%), unknown (32.6%), or no HPV vaccination (34.8%). Most patients in their index pregnancy have never used substances (79.8%); some had formerly (12.8%) or currently (7.4%) used substances. Patient demographics are summarized in Table 1.

#### Pregnancy related demographics and outcomes

In the index pregnancy, the mean (SD) gravidity was 2.9 (2.01). Mean gravidity of the second and third pregnancies was 3.9 (2.82) and 3.5 (0.82) for persistently positive cHPV testing (cHPV+) and 3.6 (1.93), 5.4 (2.32), for negative cHPV testing (cHPV−), with no significant differences across the cHPV + and cHPV− groups within each pregnancy. There was no difference in parity across the groups as well (Table 2).

**Figure 2:**
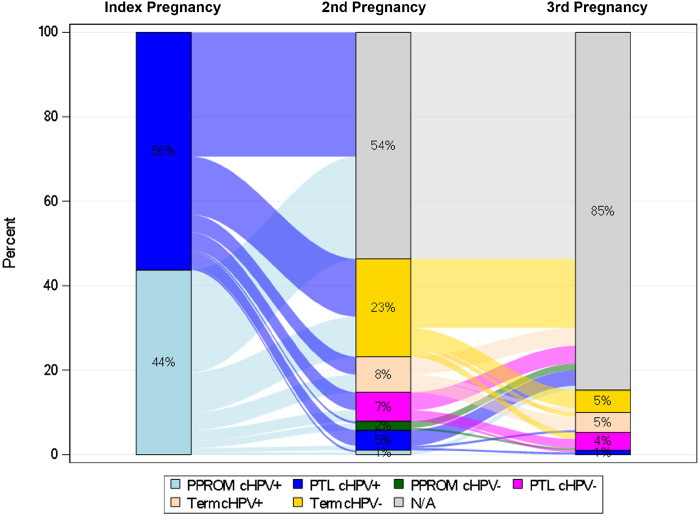
Flow of patients among index pregnancy, 2^nd^ pregnancy, and 3^rd^ pregnancy outcomes

During the index pregnancy, 83/190 (43.7%) and 107/190 (56.3%) of patients experienced PPROM and PTL, respectively. 21/74 (28.4%) patients experienced PPROM or PTL in any subsequent pregnancy (Fig. 2). Among patients with persistent cHPV+, 2/27 (7.4%) and 9/27 (33.3%) experienced PPROM and PTL in the second pregnancy; 2/11 (18.2%) and 0/11 (0.0%) experienced PPROM and PTL in the third pregnancy. Conversely, 4/62 (6.3%) and 13/62 (20.6%) cHPV− patients experienced PPROM and PTL in the second pregnancy and 8/19 (42.1%) and 0/19 (0.0%) in the third pregnancy (Fig. 2). By chi-square test or Fisher exact test as appropriate, the differences among the groups of patients and their outcomes were not significantly different

### Cervical screening and procedures

In the index pregnancy, 22/183 (12.0%) of patients had a loop electrosurgical excision procedure (LEEP) or cone 33/156 (21.2%) prior to and after the eligible index pregnancy. There was no difference in cHPV + vs cHPV− groups (Table 3), or the primary outcome of PPROM/PTL after adjusting for excisional procedures or vaccination status (**Supplemental Table 1**) across each subsequent pregnancy, as noted by Fisher test and chi-square test of multiple comparisons, respectively

Antenatal cervical cytology demonstrated no intraepithelial lesion or malignancy (NILM) (69/162, 45.7%), atypical squamous cells of undetermined significance (ASCUS) (36/162, 22.2%), low grade squamous intraepithelial lesion (LSIL) (31/162, 19.1%), atypical squamous cells of undetermined significance – cannot exclude high grade lesion (ASCH) (5/162, 3.1%), atypical glandular cells of undetermined significance (AGUS) (2/162, 1.2%), and high grade squamous intraepithelial lesion (HSIL) (13/162, 8.0%) in the index pregnancy. Upon examining subsequent pregnancies, there was no significant difference in pap cytology by chi-square test across each pregnancy by cHPV + vs cHPV− in antenatal and postpartum sampling.

The presence and persistence of cHPV and cHPV type detected in each pregnancy are shown in Table 3. Following the index pregnancy, 65/153 (42.5%) of patients demonstrated cHPV persistence > 6 months postpartum on subsequent pap smears by PCR. Subsequent pregnancy cHPV typing demonstrated no differences in initial cHPV strains between cHPV + and cHPV− groups between the initial and subsequent pregnancies. Additionally, patients with persistent cHPV did not have changes (18/27, 66.7%) or had unknown changes (4/27, 14.8%) in HPV type from the index to subsequent pregnancy.

#### Colposcopy

Most patients (138/191, 72.3%) in the index pregnancy group underwent colposcopy any time prior to or during pregnancy, during which a minority were diagnosed with no lesions via negative biopsy (36/132, 27.3%) and the remaining majority with cervical intraepithelial neoplasia (CIN) I (51/132, 38.6%), II (15/132, 38.6%), and III (30/132, 22.7%). There are no differences in the proportion of patients who received colposcopy and in the colposcopy biopsy results across all pregnancies (comparison by cHPV+) (Table 2). Those with excisional procedures before their second and third pregnancy had PTL/PPROM recurrence rates of 9/21 (42.9%) and 2/6 (33.3%). Among those with excisional procedures before their second and third pregnancy, 4/21 (19.0%) and 0/6 (0.0%) were cHPV + in the subsequent pregnancy. There were no statistically significant differences.

#### Placental pathology

In the index pregnancy, 39/162 (24.1%) of patients who had placental histology evaluation had inflammation noted (including acute chorioamnionitis, funisitis, and acute and chronic villitis). 47/90 (52.2%) and 11/30 (36.7%) of the total placentas underwent pathologic evaluation in the second, and third pregnancy, respectively. Among patients with persistently positive cHPV tests, 5/13 (38.5%) and 1/4 (25.0%) had a presence of inflammation (defined as the three pathologies above) in the subsequent second and third pregnancies, respectively. Among the cHPV− patients, 4/34 (11.8%) and 0/7 (0.0%) had a presence of inflammation in the subsequent second and third pregnancies, respectively. Although there was a higher proportion of placental inflammation in continued cHPV+ patients, this was not statistically significant. Additionally, there was no difference in evidence of placental abruption, disruption of maternal surface of placenta, and placental weight across subsequent pregnancies, analyzed by cHPV-positivity (**Supplemental Table 2**).

## Discussion

### Principal Findings

In this study, we aimed to determine the risk of recurrent adverse pregnancy outcomes among patients with persistent cHPV infection. We hypothesized that in the high-risk population with a history of PPROM or PTL, the persistence of cHPV virus from one pregnancy to another increases the risk of recurrent adverse pregnancy outcomes (primarily PPROM or PTL) in the subsequent pregnancy. While we noted a higher likelihood of recurrent PPROM/PTL in this population, this finding was not significantly different by the presence of persistent cHPV positivity. Additional key findings included increased likelihood of pathologic evidence of inflammation (chorioamnionitis, villitis, funisitis) among cHPV+ when compared to cHPV− patients, but this was not statistically significant.

## Results

We found that there were higher rates of PPROM/PTL recurrence, but not statistically significant, across the population of patients with cHPV positivity compared to those with cHPV negativity by pap smear. However, this trend was not statistically significant. This higher recurrence rate may have many contributing factors, including the performance of interval cervical procedures, predisposing demographic factors, and altered screening/surveillance once an at-risk pregnancy is identified. Our baseline rate of 41% recurrence risk for persistent cHPV positive and 27% recurrence risk for cHPV negative patients is comparable to published estimates of the baseline risk of recurrent PPROM/PTL (7–34%) among patients with unknown cervical HPV results (28–30). Notably, a meta-analysis found that risk of spontaneous PTB recurrence due to PPROM was 7% and PTL was 23% (28).

Paramount to any understanding of the recurrence risk of PPROM and PTL for patients with cHPV is understanding the impact of the performance of LEEP and cone procedures, which were commonly performed in our cohort. Many patients clear their HPV with a LEEP/cone procedure but are thought to remain at risk of spontaneous PTB due to mechanical shortening of the cervix (31, 32). While LEEP/cone procedures have been shown to increase the risk of spontaneous PTB in meta-analysis, this association did not remain once patients with a history of LEEP were compared with patients having a history cervical dysplasia but no cervical excision (33). The lack of an independent effect of excision on PTB was recently supported by a 2021 study of 1231 patients, including 634 patients with excisional procedures, noting no significant increase in PTB among patients who had a previous spontaneous PTB and large loop excision (37.0%), compared to the baseline recurrence rate in patients who had previous spontaneous PTB and no excision (28.7%) (34). Interestingly, there was no difference in rates of recurrent PPROM/PTL noted in those whose cHPV testing transitioned to negative.

Additionally, colposcopy found that CIN progression was observed in patients with persistent cHPV. Even though CIN lesions show high spontaneous regression rates postpartum, it was unknown what the consequences of persistent cHPV positivity, including progression of CIN, would be in our population (35, 36). Our study found high rates of CIN in colposcopy among patients with persistent cHPV positivity, a finding which mirrors the pathologic evolution of the viral infection (37, 38).

A final notable finding in our study was the slightly elevated rate of pathologic placental inflammation among patients with persistent cHPV in subsequent pregnancies when contrasted to those without persistent cHPV, though not statistically significant. Chorioamnionitis is known to contribute to the development of both PPROM and PTL (39). Thus, we hypothesized that it is possible that the dysregulated inflammation caused by cHPV could indirectly contribute to chorioamnionitis, leading to PPROM/PTL. Supporting this hypothesis, Slatter *et al*. found that placentas with HPV positivity were significantly associated with histological acute chorioamnionitis (10). Previous studies have noted an increased probability of chorioamnionitis among patients with a history of clinical chorioamnionitis (40). Our results were consistent with these studies with high rates of placental inflammation among all patients with spontaneous PTB, noted in groups with persistent cHPV and those who cleared their infection.

### Clinical Implications

Our study provides evidence for focused counseling for patients with persistent cHPV infection and a history of PPROM/PTL, particularly surrounding the role of HPV vaccination prior to cHPV exposure. In our study, about 2/3 of index pregnancies had no HPV vaccination or unknown HPV vaccination history. Because the persistent cHPV tests for subsequent pregnancies were associated with a significant increase in pregnancy related inflammation, such as chorioamnionitis, this provides increasing call for HPV vaccination to track and prevent disease (41, 42).

### Research Implications

Prior studies support an association between HPV infection and PTB (5–8, 11, 12), with two works in dissent (1, 43). To address this discrepancy, Niyibizi *et al*. conducted a systematic review and meta-analysis, which found a positive association between HPV and spontaneous PTB (3). Across subgroups and sensitivity analyses including but not limited to maternal age, multiple pregnancies, other genital infections, parity, obstetrical risk factors, smoking, and socioeconomic risk factors, the association between HPV and PTB remained positive.

Our work furthers this field by investigating the recurrence of adverse obstetrical outcomes with persistent cHPV. Given the risk of recurrent adverse pregnancy events, particularly placental inflammation, with persistent cHPV, further research is warranted for this patient population. Importantly, there needs to be more work done to understand if persistence of cHPV directly affects PPROM/PTL or if some other pathway leads to the association (1, 15, 17).

### Strengths and Limitations

To our knowledge, this is the first study investigating the recurrence of PPROM/PTL as adverse obstetric complications from patients with persistent cHPV. Additionally, this study includes a variety of factors in the clinical characterization of cHPV infection and treatment. Finally, this study investigates longitudinal outcomes.

Limitations of our study include a low proportion of cHPV infection in the population studied, and possible type 2 error due to an underpowered study. The 2018 prevalence of cHPV + in females age 21–39 at Mayo Clinic Rochester and the surrounding Mayo Clinic Health Systems is low at approximately 10/100 people (44, 45). A 2018 study found that the prevalence and incidence of HPV infections in the U.S. was 38.4% and 19.9%, respectively, in females 15–59 years old (46). Thus, the study of a larger population with a higher proportion of cHPV is essential before making firm conclusions on the recurrence risk of PTL and PPROM in this population. Additionally, we were unable to determine all time points of HPV infection with accuracy due to the broad time range of HPV testing, and the possibility of clearance, reactivation, reinfection or latency cannot be determined. Notably, specific other high-risk HPV test strains could not be determined.

## Conclusions

High rates of PPROM/PTL recurrence, placental inflammation, and CIN were noted among patients with persistent cHPV. We noted no difference in rates of recurrent PPROM/PTL among those whose cHPV testing transitioned to negative, independent of cervical excisional procedures and HPV vaccination status. There were high rates of placental inflammation in both patients with persistent and non-persistent cHPV, with no significant difference between groups. Before drawing clinical conclusions, further research is needed into the biological mechanism of HPV infection of the placenta and fetal membranes, and on the recurrence risk of PPROM/PTL in populations with higher baseline cHPV prevalence.

## Supplementary Material

Supplementary Files

This is a list of supplementary files associated with this preprint. Click to download.

- SupplementalTables.docx

## Figures and Tables

**Table 1:** Demographic Characteristics of Participants in Index and Second Pregnancies.

Characteristic	Index Pregnancy (N = 191)	Second Pregnancy (N = 90)
**Race, N (%)**		
White	167 (91.3)	78 (89.7)
Black	7 (3.8)	5 (5.7)
Asian	6 (3.3)	3 (3.4)
American Indian/Native American	3 (1.6)	1 (1.1)
Missing	8	3
**Ethnicity, N (%)**		
Hispanic	11 (5.8)	4 (4.4)
Non-Hispanic	180 (94.2)	86 (95.6)
**Insurance Type, N (%)**		
Private	105 (55.0)	48 (53.3)
Public (non-Medicaid)	5 (2.6)	5 (5.6)
Medicaid	79 (41.4)	36 (40.0)
None	2 (1.0)	1 (1.1)
**Education Level, N (%)**		
< High School	14 (7.4)	7 (7.8)
High School	58 (30.9)	28 (31.1)
2-year or Technical Certificate	28 (14.9)	12 (13.3)
4-year Graduate	25 (13.3)	15 (16.7)
Post-Graduate	15 (8.0)	10 (11.1)
Unknown	48 (25.5)	18 (20.0)
Missing	3	0
**Smoking Status, N (%)**		
Current	53 (28.0)	23 (25.8)
Former	44 (23.3)	19 (21.3)
Never	92 (48.7)	47 (52.8)
**Race, N (%)**		
Missing	2	1
**History of Drug Use, N (%)**		
Never	150 (79.8)	N/A
Former	24 (12.8)	N/A
Current	14 (7.4)	N/A
Missing	3	N/A
**HPV Vaccination Started, N (%)**		
Yes	61 (32.6)	N/A
No	65 (34.8)	N/A
Unknown	61 (32.6)	N/A
Missing	4	N/A

N/A: not applicable as these characteristics were collected for index pregnancy only.

**Table 2:** Pregnancy Details of Index Pregnancy and Subsequent Pregnancies.

	Index Pregnancy (N = 191)	Second Pregnancy (N = 90)		P-value	Third Pregnancy (N = 30)		P-value
		cHPV+ (N = 27)	cHPV− (N = 63)		cHPV+ (N = 11)	cHPV− (N = 19)	
**Route of delivery, N (%)**				0.1659^a^			0.6583^a^
Cesarean Section	66 (34.7%)	15 (55.6%)	20 (32.3%)		2 (18.2%)	6 (31.6%)	
Vaginal	124 (65.3%)	12 (44.4%)	42 (67.7%)		9 (81.8%)	13 (68.4%)	
Missing	1	0	1		0	0	
**Gestational Age at Delivery (days)**				0.3006^b^			0.4656^b^
N	189	27	62		11	19	
Mean (SD)	233.3 (28.26)	255.6 (24.21)	259.3 (28.21)		265.9 (10.41)	243.2 (58.30)	
Median	242	260	267.5		271	260	
Range	121.0, 258.0	161.0, 280.0	141.0, 287.0		245.0, 276.0	40.0, 279.0	
**Group Type, N (%)**				0.6929^a^			0.4469^a^
PPROM	83 (43.7%)	2 (7.4%)	4 (6.3%)		2 (18.2%)	8 (42.1%)	
Preterm labor	107 (56.3%)	9 (33.3%)	13 (20.6%)		0 (0.0%)	0 (0.0%)	
Normal	0 (0.0%)	16 (59.3%)	45 (71.4%)		9 (81.8%)	11 (57.9%)	
Missing	1	0	1		0	0	
**Fetal growth restriction, N (%)**				0.6151^a^			0.6583^a^
No	155 (90.6%)	23 (88.5%)	52 (94.5%)		9 (100.0%)	15 (93.8%)	
Yes	16 (9.4%)	3 (11.5%)	3 (5.5%)		0 (0.0%)	1 (6.3%)	
Missing	20	1	8		2	3	
**Gravidity**				0.9111^a^			0.0989^a^
N	191	27	62		11	19	
Mean (SD)	2.9 (2.01)	3.9 (2.82)	3.6 (1.93)		3.5 (0.82)	5.4 (2.32)	
Median	2	3	3		3	5	
Range	1.0, 13.0	2.0, 16.0	2.0, 10.0		3.0, 5.0	3.0, 11.0	
**Parity: Mean (SD)**							
Term	0.96 (1.30)	1.52 (1.83)	1.29 (0.99)	0.4553^a^	1.91 (1.16)	2.16 (1.27)	0.6102^a^
Preterm	1.20 (0.60)	1.56 (0.79)	1.37 (0.57)	0.2238^a^	1.45 (0.78)	1.53 (0.75)	0.8122^a^
Abortions and Miscarriages	0.69 (1.09)	0.70 (1.01)	0.87 (1.33)	0.567^a^	0.18 (0.39)	1.63 (1.69)	0.0115^a^
Living Children	2.17 (1.42)	3.07 (1.82)	2.68 (1.17)	0.229^a^	3.09 (0.67)	3.42 (1.31)	0.458^a^
Missing	0	0	1		0	0	
**Multiple gestation, N (%)**				0.4223^b^			0.4469^a^
Yes	21 (11.0%)	3 (11.1%)	2 (3.2%)		1 (9.1%)	0 (0.0%)	
No	170 (89.0%)	24 (88.9%)	60 (96.8%)		10 (90.9%)	18 (100.0%)	
Missing	0	0	1		0	1	

a denotes chi-square test.

b denotes Kruskal-Wallis test.

**Table 3:** Cervical Procedures, Cervical Cytology and Pathology of Index and Subsequent Pregnancies.

	Index Pregnancy (N = 191)	Second Pregnancy (N = 90)		P-value	Third Pregnancy (N = 30)		P-value
		cHPV+ (N = 27)	cHPV− (N = 63)		cHPV+ (N = 11)	cHPV− (N = 19)	
**LEEP or cone prior to eligible pregnancy, N (%)**				0.4469^a^			0.1659^a^
No	161 (88.0%)	22 (84.6%)	43 (71.7%)		11 (100.0%)	13 (68.4%)	
Yes	22 (12.0%)	4 (15.4%)	17 (28.3%)		0 (0.0%)	6 (31.6%)	
Missing	8	1	3				
**LEEP or cone after eligible pregnancy, N (%)**				0.7719^a^			0.7719^a^
No	123 (78.8%)	24 (92.3%)	54 (94.7%)		10 (90.9%)	18 (94.7%)	
Yes	33 (21.2%)	2 (7.7%)	3 (5.3%)		1 (9.1%)	1 (5.3%)	
Missing	35	1	6				
**Antenatal cervical cytology, N (%)**				0.3006^a^			0.1659^a^
NILM	74 (45.7%)	12 (48.0%)	46 (79.3%)		4 (40.0%)	14 (87.5%)	
LSIL	31 (19.1%)	4 (16.0%)	4 (6.9%)		2 (20.0%)	1 (6.3%)	
ASCUS	36 (22.2%)	5 (20.0%)	6 (10.3%)		4 (40.0%)	1 (6.3%)	
ASC-H	5 (3.1%)	1 (4.0%)	1 (1.7%)		0 (0.0%)	0 (0.0%)	
AGUS	2 (1.2%)	1 (4.0%)	0 (0.0%)		0 (0.0%)	0 (0.0%)	
HSIL	13 (8.0%)	2 (8.0%)	1 (1.7%)		0 (0.0%)	0 (0.0%)	
Missing	29	2	5		1	3	
**Antenatal HPV type, N (%)**				0.0043^a^			0.1659^a^
16	5 (3.8%)	3 (12.5%)	0 (0.0%)		0 (0.0%)	0 (0.0%)	
18	4 (3.0%)	2 (8.3%)	1 (2.4%)		1 (10.0%)	0 (0.0%)	
Other High Risk	93 (69.91/133%)	14 (58.3%)	6 (14.3%)		7 (70.0%)	2 (20.0%)	
16 and Other High Risk	1 (0.75%)	0 (0.0%)	0 (0.0%)		0 (0.0%)	0 (0.0%)	
18 and Other High Risk	2 (1.5%)	1 (4.2%)	0 (0.0%)		0 (0.0%)	0 (0.0%)	
Negative HPV test	28 (21.1%)	4 (16.7%)	35 (83.3%)		2 (20.0%)	8 (80.0%)	
Missing^b^	58	3	21		1	9	
**Postpartum cervical cytology, N (%)**				0.5258^a^			0.6425^a^
NILM	84 (53.5%)	8 (42.1%)	23 (62.2%)		3 (37.5%)	6 (46.2%)	
LSIL	22 (14.0%)	2 (10.5%)	2 (5.4%)		0 (0.0%)	2 (15.4%)	
ASCUS	34 (21.7%)	0 (0.0%)	0 (0.0%)		5 (62.5%)	5 (38.5%)	
ASC-H	1 (0.6%)	9 (47.4%)	10 (27.0%)		0 (0.0%)	0 (0.0%)	
AGUS	1 (0.6%)	0 (0.0%)	0 (0.0%)		0 (0.0%)	0 (0.0%)	
HSIL	14 (8.9%)	0 (0.0%)	2 (5.4%)		0 (0.0%)	0 (0.0%)	
Missing	34	8	26		3	6	
**Postpartum HPV type, N (%)**				0.1109^a^			0.3162^a^
16	16 (11.0%)	0 (0.0%)	3 (9.1%)		0 (0.0%)	1 (8.3%)	
18	3 (2.1%)	2 (11.8%)	0 (0.0%)		1 (12.5%)	0 (0.0%)	
Other high risk	87 (59.6%)	14 (82.4%)	18 (54.5%)		6 (75.0%)	4 (33.3%)	
18 and Other high risk	1 (0.7%)	0 (0.0%)	0 (0.0%)		0 (0.0%)	0 (0.0%)	
Negative HPV test	35 (24.0%)	1 (5.9%)	12 (36.4%)		1 (12.5%)	7 (58.3%)	
Missing^b^	45	10	30		3	7	
**Evidence of HPV persistence > 6 months postpartum on subsequent pap smears, N (%)**				0.0100^a^			0.0043^a^
Yes	65 (42.5%)	13 (65.0%)	7 (19.4%)		8 (88.9%)	0 (0.0%)	
No	88 (57.5%)	7 (35.0%)	29 (80.6%)		1 (11.1%)	8 (100.0%)	
Missing	38	7	27		2	11	
**Colposcopy, N (%)**				0.7658^a^			0.6952^a^
No	53 (27.7%)	5 (18.5%)	14 (23.0%)		1 (9.1%)	3 (16.7%)	
Yes	138 (72.3%)	22 (81.5%)	47 (77.0%)		10 (90.9%)	15 (83.3%)	
Missing	0	0	2		0	1	
**Colposcopy Path Biopsy Results, N (%)**				0.3216^a^			0.4489^a^
Benign biopsy	36 (27.3%)	7 (33.3%)	15 (34.9%)		3 (33.3%)	5 (35.7%)	
CIN 1	51 (38.6%)	10 (47.6%)	13 (30.2%)		5 (55.6%)	4 (28.6%)	
CIN 2	15 (11.4%)	2 (9.5%)	3 (7.0%)		0 (0.0%)	2 (14.3%)	
CIN 3	30 (22.7%)	2 (9.5%)	12 (27.9%)		1 (11.1%)	3 (21.4%)	
Missing	6	1	4		1	1	

a denotes chi-square test.

b positivity by historic clinical notes or ICD 9/10 codes with typing not possible.

## Data Availability

The datasets used and/or analyzed during the current study are available from the corresponding author on reasonable request.

## Abbreviations

ASCUS: Atypical cells of undetermined significance
AGUS: Atypical glandular cells of undetermined significance
ASCH: Atypical squamous cells of undetermined significance - cannot exclude high grade lesion
cHPV: Cervical high-risk human papilloma virus
CIN: Cervical intraepithelial neoplasia
HSIL: High grade squamous intraepithelial lesion
HPV: Human papilloma virus
IRB: Institutional review board
LEEP: Loop electrosurgical excision procedure
NILM: no intraepithelial lesion or malignancy
PCR: Polymerase chain reaction
PPROM: Preterm premature rupture of membranes
PTL: Preterm labor
